# Supplementary material for: A UMLS-based spell checker for natural language processing in vaccine safety
Source: BMC Med Inform Decis Mak. 2007 Feb 12;7:3. doi: 10.1186/1472-6947-7-3 (PMC1805499; doi:10.1186/1472-6947-7-3)
Supplement: Additional file 13 — RAP application directory. Sets up directory for RAP (RDF application for PHP) source code [file 1472-6947-7-3-S13.gz › rap/doc/phpdoc/__filesource/fsource_dataset__datasetDatasetMem.php.html]

xml version="1.0" encoding="iso-8859-1"?


File Source for DatasetMem.php


# Source for file DatasetMem.php

Documentation is available at DatasetMem.php

```
1. <?php
2. // ----------------------------------------------------------------------------------
3. // Class: DatasetMem
4. // ----------------------------------------------------------------------------------


8. /**
9. * In-memory implementation of a RDF dataset.
10. * A RDF dataset set is a collection of named RDF graphs.
11. *
12. * <BR><BR>History:<UL>
13. * <LI>05-11-2005                : First version of this class.</LI>
14. * <LI>06-27-2005                : Support for a defaultGraph added</LI>
15. *
16. * @version  V0.9
17. * @author Daniel Westphal (http://www.d-westphal.de)
18. * @author Chris Bizer <chris@bizer.de>
19. *
20. * @package     dataset
21. * @access    public
22. ***/

24. class DatasetMem extends Dataset
25. {

27. /**
28. * Reference to a memmodel that holds the default graph
29. *
30. * @var        resource memmodel
31. * @access    private
32. */
33. var $defaultGraph;


36. /**
37. * Name of the DatasetMem
38. *
39. * @var        string
40. * @access    private
41. */
42. var $setName;

44. /**
45. * List of all NamedGraphs
46. *
47. * @var        array
48. * @access    private
49. */
50. var $graphs=array();

52. /**
53. * Constructor
54. * You can supply a Dataset name.
55. *
56. * @param string
57. * @access    public
58. */
59. function DatasetMem($datasetName = null)
60. {
61. $this->setDatasetName($datasetName);
62. $this->defaultGraph=new MemModel();
63. }

65. //    === Graph level methods ========================


69. /**
70. * Sets the Dataset name.
71. *
72. * @param  string
73. * @access    public
74. */
75. function setDatasetName($datasetName)
76. {
77. $this->setName=$datasetName;
78. }

80. /**
81. * Return the Dataset name.
82. *
83. * @return string
84. * @access    public
85. */
86. function getDatasetName()
87. {
88. return $this->setName;
89. }

91. /**
92. * Adds a NamedGraph to the set. Will replace a NamedGraph with the same name that is already in the set.
93. * @param NamedGraphMem
94. */
95. function addNamedGraph(&$graph)
96. {
97. $graphNameURI=$graph->getGraphName();
98. $this->graphs[$graphNameURI]=&$graph;
99. }

101. /**
102. * Overwrites the existting default graph.
103. *
104. * @param MemModel
105. */
106. function setDefaultGraph(&$graph)
107. {
108. $this->defaultGraph=&$graph;
109. }

111. /**
112. * Returns a reference to the defaultGraph
113. * @return Model
114. */
115. function &getDefaultGraph()
116. {
117. return ($this->defaultGraph);
118. }

120. /**
121. * Returns true, if an defaultGraph exists. False otherwise
122. * @return boolean
123. */
124. function hasDefaultGraph()
125. {
126. return ($this->defaultGraph!=null);
127. }

129. /**
130. * Removes a NamedGraph from the set. Nothing happens
131. * if no graph with that name is contained in the set.
132. * @param string
133. */
134. function removeNamedGraph($graphName)
135. {
136. unset($this->graphs[$graphName]);
137. }

139. /**
140. * Tells wether the Dataset contains a NamedGraph.
141. * @param  string
142. * @return boolean
143. */
144. function containsNamedGraph($graphName)
145. {
146. return (isset($this->graphs[$graphName])===true);
147. }

149. /**
150. * Returns the NamedGraph with a specific name from the Dataset.
151. * Changes to the graph will be reflected in the set.
152. * @param string
153. * @return NamedGraphMem or NULL
154. */
155. function &getNamedGraph($graphName)
156. {
157. if (!isset($this->graphs[$graphName])) return NULL;
158. return ($this->graphs[$graphName]);
159. }

161. /**
162. * Returns the names of the namedGraphs in this set as strings in an array.
163. * @return Array
164. */
165. function listGraphNames()
166. {
167. return array_keys($this->graphs);
168. }

170. /**
171. * Creates a new NamedGraph and adds it to the set. An existing
172. * graph with the same name will be replaced.The name of the NamedGraph to be created ; must be an URI
173. * @param  string
174. * @param  string
175. * @return NamedGraphMem
176. */
177. function &createGraph($graphName,$baseURI = null)
178. {
179. $this->graphs[$graphName]=new NamedGraphMem($graphName,$baseURI);
180. return $this->getNamedGraph($graphName);
181. }

183. /**
184. * Deletes all NamedGraphs from the set.
185. */
186. function clear()
187. {
188. $this->graphs = array();
189. }

191. /**
192. * Returns the number of NamedGraphs in the set. Empty graphs
193. * are counted.
194. * @return int
195. */
196. function countGraphs()
197. {
198. return count($this->graphs);
199. }

201. /**
202. * Returns the NamedGraph with a specific offset in the dataset.
203. * Changes to the graph will be reflected in the set.
204. * @param int
205. * @return NamedGraphMem or null
206. * @access    private
207. */
208. function &getGraphWithOffset($offset)
209. {
210. $i=0;
211. foreach ($this->graphs as $graph)
212. {
213. if (($i++)==$offset)
214. return $graph;
215. }
216. return NULL;
217. }

219. /**
220. * Returns an iterator over all {@link NamedGraph}s in the set.
221. * @return IteratorAllGraphsMem
222. */
223. function &listNamedGraphs()
224. {
225. return (new IteratorAllGraphsMem($this));
226. }

228. /**
229. * Tells wether the set contains any NamedGraphs.
230. * @return boolean
231. */
232. function isEmpty()
233. {
234. return ($this->countGraphs()==0);
235. }

237. /**
238. * Add all named graphs of the other dataset to this dataset
239. * @param Dataset
240. */
241. function addAll($otherDataset)
242. {
243. for($iterator = $otherDataset->listNamedGraphs(); $iterator->valid(); $iterator->next())
244. {
245. $current=$iterator->current();
246. $this->graphs[$current->getGraphName()]=$current;
247. };

249. if ($otherDataset->hasDefaultGraph())
250. {
251. $this->defaultGraph = $this->defaultGraph->unite($otherDataset->getDefaultGraph());
252. }
253. }

255. //    === Quad level methods ========================


259. /**
260. * Adds a quad to the Dataset. The argument must not contain any
261. * wildcards. If the quad is already present, nothing happens. A new
262. * named graph will automatically be created if necessary.
263. * @param Quad
264. */
265. function addQuad(&$quad)
266. {
267. $graphName=$quad->getGraphName();
268. if ($this->containsNamedGraph($graphName->getLabel())===false)
269. $this->createGraph($graphName->getLabel());

271. $this->graphs[$graphName->getLabel()]->add($quad->getStatement());
272. }


275. /**
276. * Tells wether the Dataset contains a quad or
277. * quads matching a pattern.
278. *
279. * @param Resource $graphName
280. * @param Resource $subject
281. * @param Resource $predicate
282. * @param Resource $object
283. * @return boolean
284. */
285. function containsQuad($graphName,$subject,$predicate,$object)
286. {
287. if($graphName!=null)
288. {
289. if ($this->containsNamedGraph($graphName->getLabel())!==true)
290. return false;

292. return ($this->graphs[$graphName->getLabel()]->findFirstMatchingStatement($subject,$predicate,$object)!=null);
293. }

295. foreach ($this->graphs as $graph)
296. {
297. if ($graph->findFirstMatchingStatement($subject,$predicate,$object)!=null)
298. return true;
299. };

301. return false;
302. }

304. /**
305. * Deletes a Quad from the RDF dataset.
306. * @param Quad
307. */
308. function removeQuad($quad)
309. {
310. $graphName=$quad->getGraphName();

312. if($graphName!=null)
313. {
314. if ($this->containsNamedGraph($graphName->getLabel())!==true)
315. return;

317. return ($this->graphs[$graphName->getLabel()]->remove($quad->getStatement())!=null);

319. }
320. foreach ($this->graphs as $graph)
321. {
322. $graph->remove($quad->getStatement());
323. };
324. }

326. /**
327. * Counts the Quads in the RDF dataset. Identical Triples in
328. * different NamedGraphs are counted individually.
329. * @return int
330. */
331. function countQuads()
332. {
333. $count=0;
334. foreach ($this->graphs as $graph)
335. {
336. $count+=$graph->size();
337. }
338. return $count;
339. }

341. /**
342. * Finds Statements that match a quad pattern. The argument may contain
343. * wildcards.
344. * @param Resource or Null
345. * @param Resourceor Null
346. * @param Resource or Null
347. * @param Resource or Null
348. * @return Iterator
349. */
350. function &findInNamedGraphs($graph,$subject,$predicate,$object)
351. {
352. if ($graph!=null)
353. {
354. $findGraph=&$this->getNamedGraph($graph->getLabel());
355. if($findGraph==null)
356. $findGraph=new MemModel();

358. return $findGraph->iterFind($subject,$predicate,$object);
359. }

361. return new IteratorFindQuadsMem($subject,$predicate,$object,$this->listNamedGraphs(),true);
362. }

364. /**
365. * Finds Statements that match a pattern in the default Graph. The argument may contain
366. * wildcards.
367. * @param Resource or Null
368. * @param Resource or Null
369. * @param Resource or Null
370. * @return Iterator
371. */
372. function &findInDefaultGraph($subject,$predicate,$object)
373. {
374. return $this->defaultGraph->iterFind($subject,$predicate,$object);
375. }
376. }
377. ?>
```

Documentation generated on Thu, 7 Jul 2005 13:41:14 +0200 by phpDocumentor 1.3.0RC3
